# Physiological Responses to Auditory, Visual, and Auditory Imagery Triggers in Misophonia

**DOI:** 10.1111/psyp.70390

**Published:** 2026-09-02

**Authors:** Xuan Yang, Sewon Oh, Douglas H. Wedell, Svetlana V. Shinkareva

**Affiliations:** ^1^ Department of Psychology University of South Carolina Columbia South Carolina USA; ^2^ Institute for Mind and Brain, University of South Carolina Columbia South Carolina USA

## Abstract

Misophonia is characterized by decreased tolerance to specific trigger sounds, yet the underlying physiological mechanisms remain poorly understood. Misophonic responses are also often reported to be elicited not only by the auditory triggers themselves, but also by visual cues associated with those sounds. However, direct empirical evidence for visual cues associated with auditory triggers remains limited. Moreover, it is not known if misophonic responses can be elicited in the absence of any sensory input, such as during auditory mental imagery. The present study systematically examined physiological responses to misophonic trigger stimuli across auditory and visual sensory modalities, as well as auditory imagery, using multi‐channel psychophysiological measures and both trial‐wise and time‐resolved approaches. Individuals with misophonia and matched control participants were exposed to trigger, generally aversive, and non‐aversive stimuli presented in auditory, visual (silent video), and auditory imagery conditions. Physiological responses were recorded across five channels, including electromyographic activity over corrugator supercilii (EMGc) and zygomaticus major (EMGz) muscles, electrodermal activity (EDA), heart rate (HR), and fingertip skin temperature. Compared with generally aversive stimuli, auditory trigger sounds elicited significantly greater physiological responses in individuals with misophonia than in control participants, characterized by increased EMGc, EMGz, EDA, and HR, indicating heightened affective and autonomic engagement beyond general aversiveness. Silent visual trigger cues also evoked greater trigger‐specific physiological responses, although these effects were observed in fewer physiological channels (primarily EMGc and HR). Auditory mental imagery produced weaker group‐level physiological effects, with significant differentiation limited to EDA. Across all three modalities, greater trial‐wise subjective distress was consistently associated with greater EMGc, EMGz, and EDA responses, as well as greater HR responses in the auditory and visual modalities, indicating a close correspondence between physiological activation and the subjective experience of misophonic distress. In contrast, physiological overactivation was not significantly associated with overall misophonia severity or auditory imagery ability. Together, these findings provide the most comprehensive characterization to date of peripheral physiological responses in misophonia across auditory and visual perception, as well as auditory imagery. They demonstrate that misophonia is characterized by exaggerated affective and autonomic physiological responses to trigger sounds, extend this response profile to silent visual cues associated with trigger sounds, and provide initial evidence that auditory mental imagery can engage selected components of the misophonic response.

## IMPACT STATEMENT

This study provides the first comprehensive characterization of physiological responses to misophonic triggers across auditory and visual perception, as well as auditory mental imagery. The findings demonstrate heightened physiological responses to misophonic triggers beyond those evoked by generally aversive stimuli and position misophonia as a disorder of maladaptive salience attribution.

## 1 Introduction

Misophonia is characterized by decreased tolerance to specific sounds, known as triggers, as well as to stimuli associated with those sounds (Swedo et al. 2022), and is estimated to affect approximately 5% of the population (Dixon et al. 2024). The term misophonia was first introduced by Pawel J. Jastreboff in 2001 to distinguish the condition from other forms of decreased sound tolerance, such as hyperacusis and phonophobia (Jastreboff and Jastreboff 2001). Individuals with misophonia typically experience abnormally strong negative reactions to otherwise innocuous everyday sounds, such as chewing or tapping. These reactions often involve extreme distress, disgust, and anger, and may be accompanied by impulsive or aggressive behavioral urges (Edelstein et al. 2013; Hanna et al. 2025; Jastreboff and Jastreboff 2023; Kumar et al. 2017; Rouw and Erfanian 2018; Schröder et al. 2013). Because trigger sounds are common in daily life and difficult to avoid, misophonia can lead to substantial impairments in social functioning, family relationships, and personal well‐being (Guzick et al. 2023; Rinaldi et al. 2022; Rinaldi and Simner 2025; Simner and Rinaldi 2023). Despite these clinically significant features, misophonia has not yet been included in contemporary psychiatric classification systems, and research on its neurophysiological mechanisms remains in its early stages.

A prominent theoretical account of misophonia proposes that the condition arises from abnormally strong activation within autonomic and limbic systems, leading to exaggerated emotional and physiological reactions to trigger stimuli (Jastreboff and Jastreboff 2023). Consistent with this view, a large‐scale behavioral study reported that most individuals with misophonia experience muscle tension and heart rate acceleration in response to trigger sounds, with some also reporting bodily pressure, sweating, and shortness of breath (Rouw and Erfanian 2018). Edelstein et al. (2013) demonstrated that misophonic responses to trigger sounds, relative to generally aversive stimuli, were associated with heightened skin conductance responses, a well‐established indicator of autonomic arousal (Bradley, Codispoti, Cuthbert, and Lang 2001; Bradley and Lang 2000, 2007; Codispoti et al. 2001; Palomba et al. 2000; Sato et al. 2020; Van Dooren et al. 2012). Extending these findings, Kumar et al. (2017) reported that, compared to control participants, individuals with misophonia exhibited greater heart rate acceleration and heightened galvanic skin responses to trigger sounds. This heightened physiological reactivity was associated with increased activation in the bilateral anterior insular cortex, a key region implicated in salience detection and interoceptive processing. Moreover, their results showed that the activation in bilateral anterior insula increased linearly with self‐reported distress ratings. Grossini et al. (2022) found that participants with misophonia demonstrated increased activation in the sympathetic nervous system and decreased activation in the parasympathetic nervous system in response to trigger sounds as measured by heart rate variability, whereas participants without misophonia demonstrated decreased activation in the sympathetic nervous system in response to trigger sounds. This pattern of autonomic activation was mirrored in their neuroimaging findings, which revealed heightened responses in auditory–insula–limbic circuits involved in salience processing. Together, these findings suggest that misophonic responses to trigger sounds, despite their innocuous nature for most people, are characterized by heightened activation of both the autonomic nervous system and central neural circuits, likely reflecting altered structure and connectivity within salience‐ and attention‐related networks.

More recent work offers a complementary perspective, highlighting the potential influence of experimental context on observed misophonic responses. Neacsiu et al. (2024) examined differences in autonomic activation, measured by skin conductance level, between participants with misophonia and clinical control patients with emotion dysregulation and found no significant group differences in response to individualized trigger sounds. Moreover, when participants with misophonia were instructed to down‐regulate their distress in response to trigger sounds, there was only a significant reduction in self‐reported ratings but not in physiological activation. However, participants with misophonia showed significantly reduced self‐reported distress and skin conductance level in response to trigger sounds during neurostimulation in the dorsolateral prefrontal cortex, a key region underlying emotion regulation, compared to the sham condition without neurostimulation. Siepsiak et al. (2023) found that individuals with misophonia rated trigger sounds as more negatively valenced, more arousing, and less controllable than control participants; however, physiological responses were largely similar between misophonia and non‐misophonia control groups. Group differences emerged only at specific time points, including elevated skin conductance responses and heart rate deceleration, and these physiological measures did not consistently align with self‐reported ratings. Such patterns may reflect methodological differences across studies, particularly with respect to task design and stimulus presentation. Notably, Siepsiak et al. (2023) manipulated stimulus context using congruent and incongruent audiovisual conditions in addition to audio‐only trials, which may have engaged distinct evaluative processes and moderated the coupling between self‐reported experience. Similarly, the inclusion of explicit emotion‐regulation demands in Neacsiu et al. (2024) may have shifted the task away from assessing pure emotional reactivity, thereby attenuating observable physiological activation.

Although misophonia is defined by decreased tolerance to sounds, misophonic reactions are not necessarily limited to trigger sounds per se. The recent consensus definition emphasized that misophonia may also be triggered by stimuli associated with such sounds, including cues from other sensory modalities (Swedo et al. 2022). Despite this acknowledgment, very few studies have empirically examined misophonic responses to non‐auditory triggers. Dozier and Morrison (2017) found that visual cues associated with trigger sounds (e.g., watching someone chew without hearing the sound) could evoke physical responses similar to those elicited by auditory triggers. In contrast, Edelstein et al. (2013) found that silent videos of trigger‐related actions did not produce the same heightened skin conductance responses observed during auditory or audiovisual trigger presentations. These mixed findings highlight the need for systematic investigation of how different sensory modalities contribute to misophonic responses (Jager et al. 2020; Johnson et al. 2013).

Another emerging characteristic of misophonia is enhanced auditory mental imagery ability. Simner et al. (2021) reported that individuals with misophonia tend to exhibit more vivid auditory imagery compared to controls. However, no study to date has directly tested whether mental imagery of trigger sounds—presented in the absence of external sensory input—can elicit misophonic distress or physiological responses. This question is particularly relevant given extensive evidence from other affective disorders, such as phobia, anxiety disorder, and post‐traumatic stress disorder (PTSD), demonstrating that affective imagery alone can evoke robust physiological reactions. In these conditions, imagery of feared or aversive stimuli has been shown to induce heart rate acceleration, increased electrodermal activity (EDA), and enhanced corrugator supercilii muscle activity, reflecting activation of defensive motivational systems (Bradley, Codispoti, Cuthbert, and Lang 2001; Bradley and Lang 2007). Although most prior studies have focused on visual mental imagery, growing evidence indicates that mental imagery is inherently multisensory (Engen et al. 2018; Sulfaro et al. 2024) and that participants can selectively attend to a specific sensory modality when instructed to do so (Yang et al. 2026). To the best of our knowledge, no study to date has directly examined whether individuals with misophonia experience self‐reported distress or physiological misophonic responses during mental imagery of their trigger sounds in the absence of external sensory input. Addressing this gap is essential for understanding the sensory and cognitive mechanisms of misophonia and may inform the development of imagery‐based interventions.

The present study aimed to systematically investigate physiological responses to misophonic trigger stimuli across auditory and visual sensory modalities, as well as auditory imagery, henceforth referred to as modality, using five physiological measures: corrugator supercilii electromyography (EMGc), zygomaticus major electromyography (EMGz), EDA, heart rate (HR), and fingertip skin temperature (SKT). EMGc potentiation, reflecting brow tightening, is a robust indicator of aversive processing (Bradley, Codispoti, Cuthbert, and Lang 2001; Bradley and Lang 2000; Brown and Schwartz 1980; Codispoti et al. 2001, 2008; Oh et al. 2025; Sato et al. 2020; Schwartz et al. 1980; Yang et al. 2026), whereas EMGz activity, associated with lip corner movement, is linked to both positive affect during smiling and negative affect disgust‐related expressions (Bradley and Lang 2000, 2007; Codispoti et al. 2008; Grewe et al. 2007; Larsen et al. 2003; Oh et al. 2025; Yang et al. 2026). Increases in EDA and decreases in fingertip skin temperature are markers of heightened autonomic arousal (Sato and Kochiyama 2022; Yang et al. 2026), and heart rate acceleration is commonly observed during highly arousing aversive processing and aversive mental imagery (Bradley, Codispoti, Cuthbert, and Lang 2001; Bradley and Lang 2007; Lang et al. 1983).

We use the term physiological overactivation to refer specifically to heightened physiological responses to misophonic trigger stimuli beyond those elicited by generally aversive stimuli in misophonia. We expect this overactivation to be present in the misophonia group and, more importantly, to be greater than the corresponding trigger‐versus‐aversive difference in the control group. Specifically, we formulated the following hypotheses:

Hypothesis 1 *Participants with misophonia will report experiencing significantly greater distress when processing trigger stimuli compared to aversive stimuli across all three modalities*.

Hypothesis 2 *Compared with control participants, individuals with misophonia will exhibit greater physiological differentiation between misophonic trigger stimuli and generally aversive stimuli across all three modalities. Specifically, trigger stimuli are expected to elicit greater EMGc, EMGz, EDA, and HR responses, together with lower fingertip SKT, than generally aversive stimuli in the misophonia group, and this trigger‐*versus*‐aversive difference will be larger than that observed in control participants. For the auditory imagery modality, however, HR is not expected to significantly differentiate trigger from aversive stimuli, as imagined trigger sounds may not elicit sufficient action‐preparation‐related cardiovascular activation*.

Hypothesis 3 *For participants with misophonia, the magnitude of physiological activation will vary linearly with self‐reported distress ratings, such that EMGc, EMGz, HR, and EDA will show linear increases, whereas SKT will show a linear decrease, as perceived distress increases across all three modalities*.

Hypothesis 4 *The magnitude of physiological activation to trigger stimuli, relative to generally aversive stimuli, will increase with greater self‐reported misophonia symptom severity and functional impairment*.

Hypothesis 5 *During auditory mental imagery, the magnitude of physiological activation to trigger stimuli, relative to generally aversive stimuli, will increase with higher auditory imagery ability, such that individuals with more vivid auditory imagery will exhibit stronger physiological responses to mental imagery of trigger sounds*.

## 2 Methods

### 2.1 Participants and Experimental Procedure

We recruited 18–45 years old English‐speaking participants without hyperacusis, hearing loss, or neurological conditions largely from the University of South Carolina community through flyers, on‐campus group presentations, and social media advertisements. For the misophonia group, 107 participants aged 18–45 with self‐identified misophonia were screened using online questionnaires and interviews to confirm that they (1) have either clinical or subclinical misophonia as defined by the Duke–Vanderbilt Misophonia Screening Questionnaire (DVMSQ); (2) reported auditory triggers that could be depicted in video stimuli by an identifiable visual component suggestive of the associated sound, (3) experienced triggers that were not specific to a particular individual, and (4) could tolerate 5‐s trigger presentations. Twenty‐eight participants were excluded for the following reasons: could not tolerate 5‐s trigger presentations (*n* = 3), neurological conditions (*n* = 2), no response to triggers during the interview (*n* = 5), triggers lacking a visual component (*n* = 2), and dropout (*n* = 16). Because participants were also recruited for a functional magnetic resonance imaging (fMRI) study, an additional 13 participants were excluded due to MRI contraindications. Consequently, 66 participants were invited to participate in the physiology experiment. During the in‐person visit, participants were further screened for psychopathology, hyperacusis, and hearing loss. Nine participants were additionally excluded due to elevated scores on SCL‐90‐R Paranoid Ideation or Psychoticism (T score > 70, *n* = 1), presence of hyperacusis or hearing loss (*n* = 6), evidence of feigned misophonia (*n* = 1), or failure to complete the full experiment (*n* = 1). As a result, the final sample consisted of 57 participants with misophonia (M_age_ = 24.28, SD_age_ = 7.27; 50 females). We added an exploratory self‐report item after data collection had begun to assess sensitivity to visual‐only triggers (e.g., repetitive foot jiggling). Among the 52 participants who completed this item, 10 (19.23%) reported such triggers.

For the control group, 63 participants were recruited largely from the University of South Carolina community through flyers, on‐campus group presentations, and social media advertisements. They completed the same online questionnaires and interviews as the misophonia group but were screened using control‐specific eligibility criteria. Specifically, participants were included if they (1) answered “No” to the binary screening item of DVMSQ assessing whether they are extremely bothered by certain sounds even when they are not loud; (2) confirmed that they could tolerate sounds commonly categorized as misophonic triggers and did not identify those sounds as personally triggering; (3) were matched to participants with misophonia on age (within 3 years), biological sex, and handedness. Seventeen participants were excluded because they reported common misophonic trigger sounds as extremely annoying during the screening interview (*n* = 6), had MRI contraindications (*n* = 8), or dropout (*n* = 3). Out of the 46 participants who were invited to the physiology experiment, two participants were excluded due to failure to complete the full experiment, and one was excluded because excessive body motion resulted in unusable physiological data. As a result, the final sample consisted of 43 control participants (M_age_ = 23.42, SD_age_ = 6.33; 36 females).

### 2.2 Stimuli

The stimuli consisted of 5‐s sounds, 5‐s silent video clips, and auditory mental imagery prompts. Stimuli varied across three conditions: generally aversive stimuli, generally non‐aversive stimuli, and individual‐specific trigger stimuli tailored to each participant with misophonia. The audiovisual clips were recorded in‐house using handheld devices. All clips were trimmed to a 4:3 aspect ratio (640 × 480 pixels) and a fixed duration of 5 s. Audio tracks were extracted from the original audiovisual clips and converted to monophonic format. A researcher listened to each audio and manually applied noise reduction using Audacity 3.6.3 to remove the background noise while keeping the quality of the target sound unchanged. These audios were then normalized to a uniform loudness level of −16 loudness units relative to full scale (LUFS) using Audacity. For the aversive and non‐aversive stimuli, validation experiments were conducted on separate samples to select the stimuli that maximally differ on aversiveness ratings for both auditory, *t*(70) = 23.94, *p* < 0.001, 95% CI [6.39, 7.55], and visual components, *t*(70) = 25.00, *p* < 0.001, 95% CI [5.81, 6.81], between aversive and non‐aversive conditions, resulting in 36 unique exemplars per condition. An example of an aversive stimulus is “a woman's high pitch scream,” which was used as the auditory prompt, with the corresponding screaming sound presented as the auditory stimulus and the corresponding visual component presented as the visual stimulus. Similarly, an example of a non‐aversive stimulus is “peaceful wind chimes”, which was used as the auditory prompt, with the corresponding wind chimes sounds presented as the auditory stimulus and the corresponding visual component presented as the visual stimulus.

For the trigger condition, we developed a Misophonia Audiovisual Trigger Archive (MATA; Oh et al. 2026), which includes stimuli spanning broad categories listed in DVMSQ that have clear visual components. These stimuli were generated following the same procedure as the aversive and non‐aversive sets. One of the authors then conducted an online interview with each participant with misophonia to review their triggers and select relevant stimuli from the archive. When the existing stimuli were not sufficient to elicit an individual's trigger response, additional individualized trigger stimuli were created. We then applied the same procedure to extract the auditory components for the audio condition as that used for the aversive and non‐aversive stimuli. All aversive and non‐aversive stimuli were presented to participants with misophonia during the interview, and any stimuli identified as triggers by the participant were excluded from those sets. Considering that we aimed to generate 36 stimuli per condition and modality for a follow‐up fMRI study, some stimuli were reused when an insufficient number of stimuli per condition remained after these exclusions.

The imagery prompts were short phrases designed to elicit auditory mental imagery. The aversive and non‐aversive imagery prompts were specifically phrased to describe our audiovisual stimuli. In contrast, the trigger imagery prompts were phrased to generally describe the trigger stimuli with variant wording of the action or the object (e.g., “Someone chewing a mouthful of salad” and “Someone chewing gummy bears with an open mouth”). The corresponding chewing sounds and visual scenes were used for the auditory and visual stimuli, respectively.

For the current physiology study, the final stimulus sets were selected separately for each participant with misophonia. For the aversive and non‐aversive conditions, selection began from the common pools of 36 validated stimuli described above. However, any stimulus identified as a personal trigger during the interview was removed from its original condition and added to that participant's candidate trigger pool, such that the eligible aversive and non‐aversive pools could differ across participants. Candidate trigger pools were individually constructed based on each participant's reported trigger categories and included multiple exemplars varying in specific objects or contexts when possible. After these participant‐specific candidate pools were established, 20 stimuli were randomly selected from each condition for each modality. The resulting stimulus set for each participant with misophonia was also presented to their matched control participant. Thus, stimulus sets could differ across matched pairs, whereas participants within a matched pair received the same stimuli. Some participants were presented with repeated stimuli either as a result of the randomization procedure or because they reported sensitivity to only a limited set of trigger stimuli. Across participants, an average of 10.26 out of the 180 trials (5.7%) were repeated (SD = 8.00, range = 0–35). Overall, 92 out of 100 participants experienced at least one repeated trial.

### 2.3 Experimental Design

**FIGURE 1:**
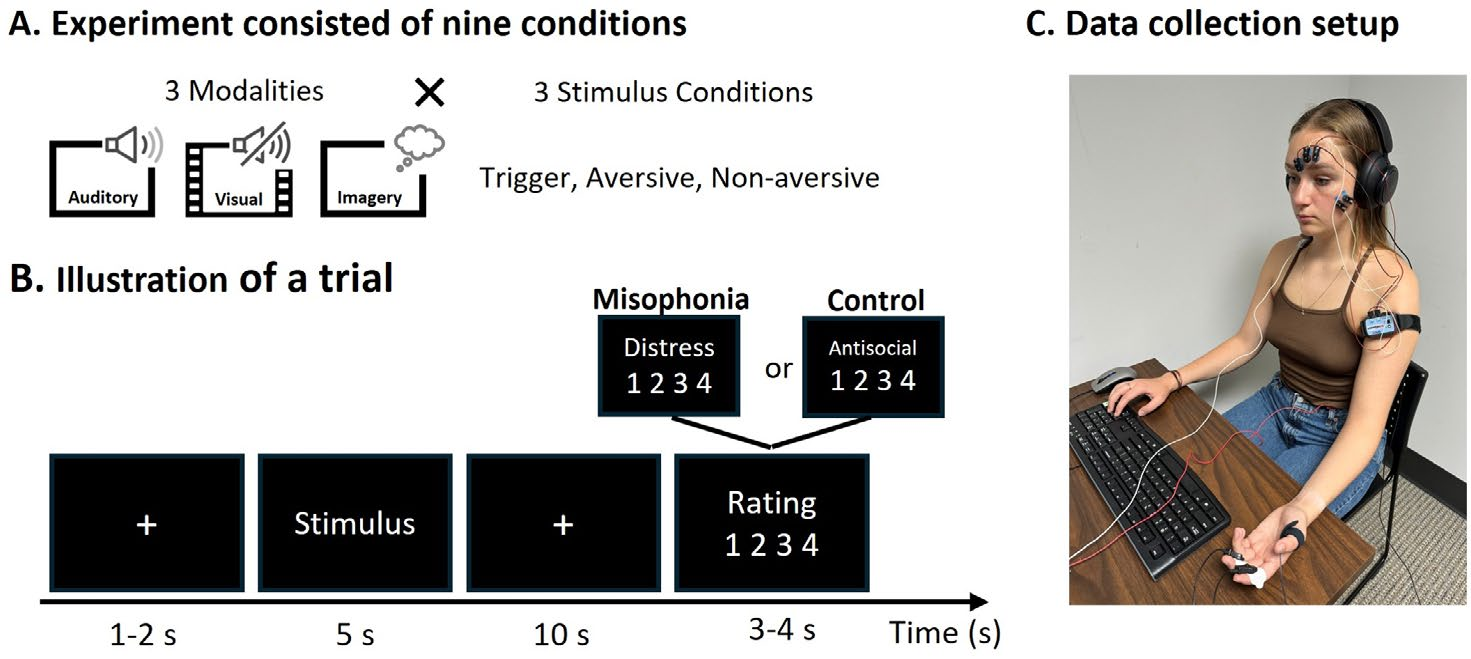
Experiment design (A and B) and data collection setup (C). Image was used with permission.

To examine the physiological mechanisms underlying trigger response across modalities, we adopted a 2 (Group: misophonia and control) × 3 (Condition: trigger, aversive, and non‐aversive) × 3 (Modality: auditory, visual, and imagery) factorial design, with Group as a between‐subject factor and Condition and Modality as within‐subject factors. The experiment included 180 stimuli in total, comprising 20 stimuli for each Condition × Modality combination. The stimuli were presented in 12 blocks, with four blocks for each modality. Each block consisted of 15 stimuli (5 stimuli per condition) from a single modality presented in a random order. Each trial was designed to last 20 s to ensure enough time for the activation and recovery of physiological responses. Each trial began with a 1–2 s fixation cross, followed by a 5 s stimulus presentation, another fixation cross presented for 10 s, and a rating screen for 3–4 s (Figure 1). All texts were displayed in white against a black background. Participants with misophonia rated each stimulus for perceived distress (“how distressing the stimuli were”), whereas control participants—who were not expected to experience distress—rated each stimulus for perceived antisociality (“how much you want to move away from the stimulus source”), using a 4‐point scale ranging from 1 (*not at all*) to 4 (*extremely*), adapted from the experimental paradigm used in Kumar et al. (2017). For the auditory and visual trials, participants were instructed to “pay attention to what you see or hear”. For the auditory mental imagery trials, participants were instructed to “imagine the sounds as vividly as possible, as if you can hear them with your mind's ear”. During the 10‐s post‐stimulus fixation period, participants were instructed to “keep your eyes on the fixation cross and relax”. Participants' facial expressions were recorded using a webcam mounted above the computer monitor.

### 2.4 Physiological Data Acquisition

The physiological responses were recorded across five channels using a BIOPAC MP150 system (Biopac Inc., Goleta, CA, USA), including EMGc, EMGz, EDA, HR, and fingertip SKT. During the initial phase of data collection, physiological signals were acquired at a sampling rate of 200 Hz for seven participants with misophonia and at 2000 Hz for two participants with misophonia, across all channels. Based on these initial recordings, the protocol was subsequently optimized to use a sampling rate of 1000 Hz for the remaining participants with misophonia (*N* = 48) and all control participants (*N* = 43). This sampling rate provides sufficient temporal resolution for physiological signal analysis while avoiding the aliasing associated with high‐frequency signals such as EMG.^1^ Acquisition procedures, including amplifier settings, electrodes, and channel‐specific recording parameters, are described in detail in our prior studies (Oh et al. 2025; Yang et al. 2026).

### 2.5 Preprocessing and Data Reduction

Physiological signals were preprocessed using Acqknowledge 5.0 (Biopac Inc., Goleta, CA, USA) and in‐house Python scripts. Raw EMG signals were band‐stop filtered at 60 Hz to remove line noise, band‐pass filtered (20–495 Hz), rectified, and smoothed using a 250‐ms moving window. Phasic EDA signals were calculated online in Acqknowledge by applying a 0.05‐Hz high‐pass filter to the tonic EDA signals. The resulting phasic signals were smoothed using a centered moving average window (50 samples) and rectified. R‐peaks were automatically detected from the raw ECG waveform using Acqknowledge. QRS complexes that were not detected or were affected by motion artifacts were manually inspected and corrected. Raw SKT signals were smoothed using a centered moving average window (50 samples).

Data reduction and feature extraction were subsequently performed in Python using two complementary approaches: a trial‐wise analysis and a time‐resolved epoch analysis. For the trial‐wise analysis, physiological responses were summarized over a 15‐s analysis window for each trial, consisting of 5‐s of stimulus presentation followed by 10‐s of post‐stimulus fixation. For EMGc, EMGz, and EDA, the area under the curve (AUC) was computed within this window, yielding a single value per trial and channel. A log_10_ transformation was applied to AUC values to reduce non‐normality. Baseline correction was performed by computing AUC values from a 1‐s pre‐stimulus interval for each trial and subtracting these baseline values from the corresponding post‐onset responses. For HR and SKT, mean values were computed over the 15‐s analysis window for each trial, and mean values from the 1‐s pre‐stimulus interval were subtracted as baseline correction.

For the time‐resolved epoch analysis, each 15‐s trial was segmented into 150 consecutive bins of 0.1‐s. Within each bin, the same feature extraction procedures as in the trial‐wise analysis were applied. Specifically, preprocessed EMGc, EMGz, and EDA signals were summarized using the AUC and log‐transformed, whereas HR and SKT signals were summarized using the mean signal amplitude. Baseline correction was applied to all channels by subtracting trial‐specific baseline values computed from the pre‐stimulus interval immediately preceding each trial. This procedure yielded time‐resolved physiological data at 10 Hz, with 150 time points per trial, channel, and participant.

### 2.6 Self‐Report Individual Differences Measures

The Duke‐Vanderbilt Misophonia Screening Questionnaire (Williams et al. 2022; DVMSQ) was used to screen for misophonia. Participants first answered a binary screening item assessing whether participants are extremely bothered by certain sounds even when they are not loud. Participants who responded “No” did not complete the remainder of the questionnaire. Those who responded “Yes” completed the remaining sections, including an open‐ended trigger‐sound question, and 18 items assessing the frequency and severity of misophonic symptoms (5‐point Likert scale: 0 = *Never/Not at all* to 4 = *Very often/An extreme amount*). A total score was also calculated by summing these items (except for Item 3), with higher scores reflecting greater symptom severity. Clinically significant misophonia and sub‐clinical misophonia are identified according to the published scoring criteria. In our study, all control participants responded “No” to the binary item and therefore did not complete the remaining questionnaire. In contrast, all participants in the misophonia group responded “Yes” and met the criteria for either *clinically significant* or *subclinical misophonia*. DVMSQ shows good internal consistency for the total score ($\omega_{T}$ = 0.98).

The Symptom Checklist‐90‐Revised (SCL‐90‐R; Derogatis 2011) was used to assess psychopathology. Participants rated 90 symptoms experienced during the past seven days on a 5‐point Likert scale (0 = *Not at all* to 4 = *Extremely*). Scores were calculated for the nine symptom dimensions and three global indices according to standard procedures, with higher scores indicating greater psychological distress. The SCL‐90‐R demonstrates good to excellent internal consistency (*α* = 0.76–0.88 across subscales; *α* = 0.97 for the Global Severity Index; Lignier et al. 2024). Because the study did not include clinical procedures for suicide risk assessment, items assessing suicidal ideation (Items 15, 59, and 63) were omitted, and all scores were calculated from the remaining items.

The Selective Sound Sensitivity Syndrome Scale (S‐Five; Vitoratou et al. 2023) was used to assess individual differences in misophonia severity across five dimensions, including Externalizing, Internalizing, Impact, Outburst, and Threat. Participants rated 25 items on an 11‐point scale (0 = *Not at all true* to 10 = *Completely true*). Total scores were calculated by summing all items, with higher scores indicating greater misophonia severity. The S‐Five has good internal consistency (Cronbach's *α* > 0.83 for the total score and all subscales).

The Vividness subscale of the Bucknell Imagery Scale (BAIS‐V; Halpern 2015) was used to measure the vividness of auditory imagery. Participants imagined sounds in 14 everyday scenarios and rated image vividness on a 7‐point scale (1 = *No image is present at all* to 7 = *The image is as vivid as the actual sound*). Higher scores indicate more vivid auditory imagery. The BAIS‐V demonstrates good internal consistency (Cronbach's *α* = 0.83).

The Vividness of Visual Imagery Questionnaire (VVIQ; Marks 1973) was used to measure the vividness of visual imagery. Participants imagined scenes in 16 everyday scenarios and rated visual imagery vividness on a 5‐point scale (1 = *Perfectly clear and as vivid as normal vision* to 5 = *No image at all, you only “know” that you are thinking of the object*). Lower scores on the VVIQ reflect greater vividness of visual imagery. This measure shows high internal reliability with a split‐half reliability of 0.85.

The Affect Intensity Measure (AIM; Larsen and Diener 1987) was used to assess individual differences in the intensity of emotional experiences. This questionnaire consists of 40 items describing typical emotional reactions to everyday events. Participants rated 40 items on a 6‐point Likert scale (1 = *Never* to 6 = *Always*), with higher scores indicating greater affect intensity. This measure shows high internal consistency with Cronbach's *α* coefficients exceeding 0.90.

The Emotion Regulation Questionnaire (ERQ; Gross and John 2003) was used to assess habitual use of cognitive reappraisal (ERQ‐CR) and expressive suppression (ERQ‐ES). The ERQ consists of 10 items. Participants rated 10 items on a 7‐point Likert scale (1 = *Strongly disagree* to 7 = *Strongly agree*). Subscale scores were calculated separately for cognitive reappraisal and expressive suppression, with higher scores indicating more frequent use of each strategy. This questionnaire shows acceptable to good internal consistency, with Cronbach's *α* ranging from 0.75 to 0.80 for cognitive reappraisal and from 0.68 to 0.76 for expressive suppression.

### 2.7 Analysis

Measure‐specific exclusions were applied prior to analysis. EMGc data were excluded for seven participants with misophonia whose data were acquired at a low sampling rate (200 Hz), and EMGz data were excluded for nine participants (seven participants with misophonia acquired at 200 Hz, as well as one with misophonia and one control participant due to technical difficulties). Behavioral questionnaire data were excluded on a measure‐specific basis when responses contained missing values. Final sample sizes were *n* = 93 (50 misophonia, 43 control) for EMGc; *n* = 91 (49 misophonia, 42 control) for EMGz; *n* = 100 (57 misophonia, 43 control) for EDA, HR, SKT, and behavioral distress/antisocial ratings; *n* = 98 (56 misophonia, 42 control) for S‐Five; *n* = 97 (55 misophonia, 42 control) for the BAIS‐V; *n* = 95 (55 misophonia, 40 control) for the VVIQ, *n* = 98 (56 misophonia, 42 control) for ERQ‐CR, *n* = 100 (57 misophonia, 43 control) for ERQ‐ES, and *n* = 92 (52 misophonia, 40 control) for AIM.

For each physiological channel, outlier detection was performed using the trial‐wise data aggregated across the 15‐s analysis window. Within each participant, trials exceeding ±3 SD from the participant's mean were removed. Missing responses and responses faster than 200 ms for distress and antisocial ratings were excluded. The proportions of excluded trials were 1.62% for EMGc, 2.09% for EMGz, 0.41% for EDA, 0.90% for HR, 1.10% for SKT, and 0.71% for distress/antisocial ratings. The same trials were also excluded from the epoch analyses.

Although we developed participant‐specific trigger stimuli, it could not be guaranteed that every stimulus assigned to the trigger condition would successfully evoke a misophonic response in that participant. Accordingly, trials that participants with misophonia rated as “not distressing at all” were treated as instances in which the intended trigger manipulation was unsuccessful and were therefore excluded from data analysis.^2^ The corresponding trials were also excluded from the matched control participants so that the same trials contributed to the comparison between groups. On average, 6.46 out of 60 trigger trials were removed across three modalities per participant: 1.11 for auditory, 2.54 for visual, and 2.81 for mental imagery.

To test whether trigger stimuli are affectively distinct in misophonia (H1), we conducted separate two‐way repeated‐measures ANOVAs for the misophonia and control groups with condition and modality as within‐participant factors. Self‐reported distress and antisocial ratings were analyzed separately as the dependent variables. Planned comparisons tested trigger versus aversive stimuli within each modality. One‐tailed tests were used for the misophonia group based on our directional hypotheses, whereas two‐tailed tests were used for controls. Additional post hoc one‐tailed comparisons tested the contrasts *trigger > non‐aversive* and *aversive > non‐aversive*. False Discovery Rate (FDR) correction was applied to control for multiple comparisons across the three modalities using the Benjamini–Hochberg procedure.

To examine whether misophonia is characterized by physiological overactivation to trigger, relative to generally aversive stimuli, compared with control participants (H2), we adopted two complementary analyses. First, trial‐wise physiological responses were analyzed separately for each physiological channel using mixed‐effects general linear regression (GLM) models. For each model, the trial‐wise physiological response served as the dependent variable, with group, condition, modality, and all interactions included as fixed effects and age and sex as covariates.^3^ A subject‐specific random intercept was included to account for repeated measurements.^4^ Our primary hypothesis concerned planned contrasts rather than the omnibus model effects. Specifically, within each modality, we compared the trigger‐versus‐aversive difference between the misophonia and control groups. Statistically, this corresponds to testing the simple Group × Condition (trigger vs. aversive) interaction within each modality, thereby quantifying whether physiological differentiation between trigger and aversive stimuli is greater in individuals with misophonia than in controls. All planned contrasts were specified as a priori and evaluated using one‐tailed tests. FDR correction was applied across the 15 planned tests (three modalities × five physiological channels) using the Benjamini–Hochberg procedure.

Second, to examine whether and how the temporal dynamics of physiological responses differed between participant groups, we conducted an epoch‐based analysis. This analysis complements the GLM approach and has been employed in prior studies to capture fine‐grained temporal variations in physiological responses that may not be evident in the trial‐wise analysis (Kumar et al. 2017; Oh et al. 2025; Siepsiak et al. 2023; Yang et al. 2026). For each participant, modality, and physiological channel, the time‐resolved data at 10 Hz were averaged across trials within each stimulus condition, yielding time‐series data consisting of 150 time bins (0.1 s per bin) per condition. To account for multiple comparisons simultaneously across channels and modalities, only the participants without missing values on any channels were included in this analysis (*n* = 49 for misophonia and *n* = 42 for control).

Two directional tests were conducted. First, within the misophonia group, a trigger‐minus‐aversive difference time series was calculated for each participant. At each time bin, these differences were tested against zero using a one‐sample *t*‐test, equivalent to a paired‐samples comparison testing whether responses were greater for trigger than aversive stimuli. Second, to test whether trigger‐related overactivation was greater in the misophonia group, participant‐level trigger‐minus‐aversive time series were compared between the misophonia and control groups using independent‐samples *t*‐tests. The directional alternative for this analysis was that the trigger‐minus‐aversive difference was greater in the misophonia group. This analysis was conceptually equivalent to the planned contrast of the simple Group × Condition (trigger vs. aversive) interaction within each modality.

All five physiological channels and three stimulus modalities were included simultaneously in each permutation analysis, resulting in 15 channel‐by‐modality combinations. Temporal adjacency was defined only between consecutive time bins within a given channel‐by‐modality combination. Clusters were formed from consecutive bins whose t‐values exceeded the critical value corresponding to an uncorrected one‐sided *p* < 0.05. Statistical significance was evaluated using 5000 permutations. For the analysis within the misophonia group, permutations were implemented by randomly sign‐flipping each participant's entire trigger‐minus‐aversive data vector, which is equivalent to randomly swapping the trigger and aversive labels within participants. For the between‐group analysis, complete participant‐level difference vectors were randomly reassigned between the two groups while retaining the original group sizes. In both analyses, each participant's data across all time bins, modalities, and channels were permuted as a single unit, thereby preserving their temporal and cross‐measure dependencies. For the observed data and each permutation, adjacent suprathreshold bins were grouped into temporal clusters, and a cluster‐mass statistic was calculated as the sum of the t‐values within each cluster. The largest cluster mass across all temporal clusters and all 15 channel‐by‐modality combinations was retained for each permutation to construct a joint null distribution. The family‐wise error–corrected *p* value for each observed cluster was determined relative to this maximum‐cluster distribution, with clusters considered significant at *p*_FWE_ < 0.05. Thus, the analysis jointly controlled the family‐wise error rate across time, physiological channels, and stimulus modalities while accounting for temporal dependence between adjacent observations (Maris and Oostenveld 2007; Sassenhagen and Draschkow 2019).

To test the correspondence between the physiological activation and self‐reported distress (H3), trial‐wise physiological responses were analyzed separately for each physiological channel using mixed‐effects GLM models. For each model, the trial‐wise physiological response served as the dependent variable, with distress ratings, modality, and their interaction included as fixed effects and age and sex as covariates. A subject‐specific random intercept was included to account for repeated measurements. Planned tests were conducted on the simple slope within each modality. One‐tailed tests were evaluated with FDR correction across the 15 planned tests.

To examine whether individual differences in misophonia severity modulate physiological responses (H4), all participants were analyzed using mixed‐effects GLM models separately for each physiological channel. For each model, the trial‐wise physiological response served as the dependent variable, with stimulus condition (trigger, aversive, and non‐aversive), S‐Five total score, and their interaction included as fixed effects and age and sex as covariates. A subject‐specific random intercept was included to account for repeated measurements. Planned contrasts compared the S‐Five slopes between the trigger and aversive conditions to determine whether symptom severity predicted greater trigger‐specific physiological responses. One‐tailed tests were evaluated with FDR correction across the 15 planned tests.

To examine whether the physiological overactivation in participants with misophonia is associated with higher auditory imagery ability (H5), trial‐wise physiological responses from participants with misophonia were analyzed using mixed‐effects GLM models separately for each physiological channel. For each model, the trial‐wise physiological response served as the dependent variable, with BAIS‐V, condition, and their interaction included as fixed effects, controlling for age, sex, and VVIQ, along with a subject‐specific random intercept. Planned contrasts compared the BAIS‐V slopes between the trigger and aversive conditions to determine whether auditory mental imagery ability predicted greater trigger‐specific physiological responses. One‐tailed tests were evaluated with FDR correction across the 15 planned tests.

## 3 Results

### 3.1 Group Differences Between Participants With Misophonia and Control Participants in Self‐Reported Questionnaires

As expected from the matching procedure, the misophonia and control groups did not differ significantly in age. Participants with misophonia scored significantly higher than control participants on the S‐Five total score and all five subscales (Table 1). To explore group differences in self‐reported questionnaire measures, we conducted independent‐samples *t*‐tests for each measure. Because the existing literature does not support specific hypotheses regarding psychopathological profile differences between individuals with and without misophonia, we used two‐tailed tests. FDR correction using the Benjamini–Hochberg procedure was applied across these exploratory analyses. The two groups showed largely comparable profiles across the SCL‐90‐R symptom dimensions, with no significant group differences observed for any subscale (all *p*s > 0.05), except Phobic Anxiety. On this subscale, the misophonia group reported significantly higher symptoms of phobic anxiety than the control group (Table 1).

**TABLE 1:** Demographic and individual differences measures for misophonia and control groups.

Measure	Mean_Misophonia_	SD_Misophonia_	Mean_Control_	SD_Control_	*t*	df	*p*	*p*_FDR_
Age	24.28	7.27	23.42	6.33	0.61	98	0.541	0.608
**Total (S‐Five)**	**121.42**	**40.92**	**43.43**	**14.11**	**11.65**	**90**	**< 0.001**	**< 0.001** ***
**Externalizing (S‐Five)**	**20.34**	**6.99**	**6.49**	**2.31**	**12.29**	**91**	**< 0.001**	**< 0.001** ***
**Internalizing (S‐Five)**	**21.10**	**9.82**	**7.95**	**2.76**	**8.40**	**91**	**< 0.001**	**< 0.001** ***
**Impact (S‐Five)**	**20.26**	**9.45**	**6.21**	**2.71**	**9.22**	**90**	**< 0.001**	**< 0.001** ***
**Outburst (S‐Five)**	**26.70**	**8.60**	**10.23**	**5.02**	**10.92**	**91**	**< 0.001**	**< 0.001** ***
**Threat (S‐Five)**	**24.10**	**10.66**	**10.74**	**4.99**	**7.46**	**91**	**< 0.001**	**< 0.001** ***
BAIS‐V	73.36	16.34	70.36	18.88	0.83	95	0.408	0.516
VVIQ	38.71	14.52	40.20	17.43	−0.45	93	0.655	0.683
GSI (SCL‐90)	52.81	7.88	49.34	7.86	2.04	88	0.044	0.089
SOM (SCL‐90)	50.00	8.80	47.13	6.26	1.70	88	0.093	0.160
OC (SCL‐90)	55.00	7.91	52.61	8.82	1.34	88	0.185	0.296
IS (SCL‐90)	53.06	7.33	51.34	7.57	1.07	88	0.288	0.406
DEP (SCL‐90)	52.15	7.37	51.13	8.89	0.59	88	0.558	0.608
ANX (SCL‐90)	50.69	9.03	46.32	8.21	2.33	88	0.022	0.059
HOS (SCL‐90)	53.27	7.62	49.55	6.98	2.34	88	0.021	0.059
**PHOB (SCL‐90)**	**54.13**	**7.60**	**48.55**	**6.52**	**3.61**	**88**	**0.001**	**0.002** **
PAR (SCL‐90)	47.42	7.69	45.74	7.70	1.02	88	0.313	0.417
PSY (SCL‐90)	52.77	7.65	49.11	7.62	2.22	88	0.029	0.069
PSDI (SCL‐90)	51.08	6.99	49.13	7.91	1.22	88	0.226	0.339
PST (SCL‐90)	52.08	7.73	48.74	7.90	1.98	88	0.050	0.093
AIM	147.60	23.21	146.20	21.01	0.29	90	0.769	0.769
ERQ‐CR	30.48	4.67	32.67	5.33	−2.13	96	0.035	0.077
ERQ‐ES	12.14	5.27	11.40	4.45	0.74	98	0.461	0.553

*Note:* Two‐tailed tests were applied for all the measures except for S‐Five. FDR correction with the Benjamini‐Hochberg procedure was applied across all tests. Rows shown in bold indicate effects that remained statistically significant after FDR correction (*p* < 0.05).

Abbreviations: AIM, Affect Intensity Measure; ANX, Anxiety; DEP, Depression; ERQ‐CR, Cognitive reappraisal; ERQ‐ES, Expressive suppression; GSI, Global severity index; HOS, Hostility; IS, Interpersonal sensibility; OC, Obsessive‐compulsive; PAR, Paranoid ideation; PHOB, Phobic‐anxiety; PSDI, Positive symptom distress index; PST, Positive symptom total; PSY, Psychoticism; SOM, Somatization.

*** *p* < 0.001.

** *p* < 0.01.

### 3.2 Self‐Reported Differences Between Trigger and Aversive Stimuli (H1)

**FIGURE 2:**
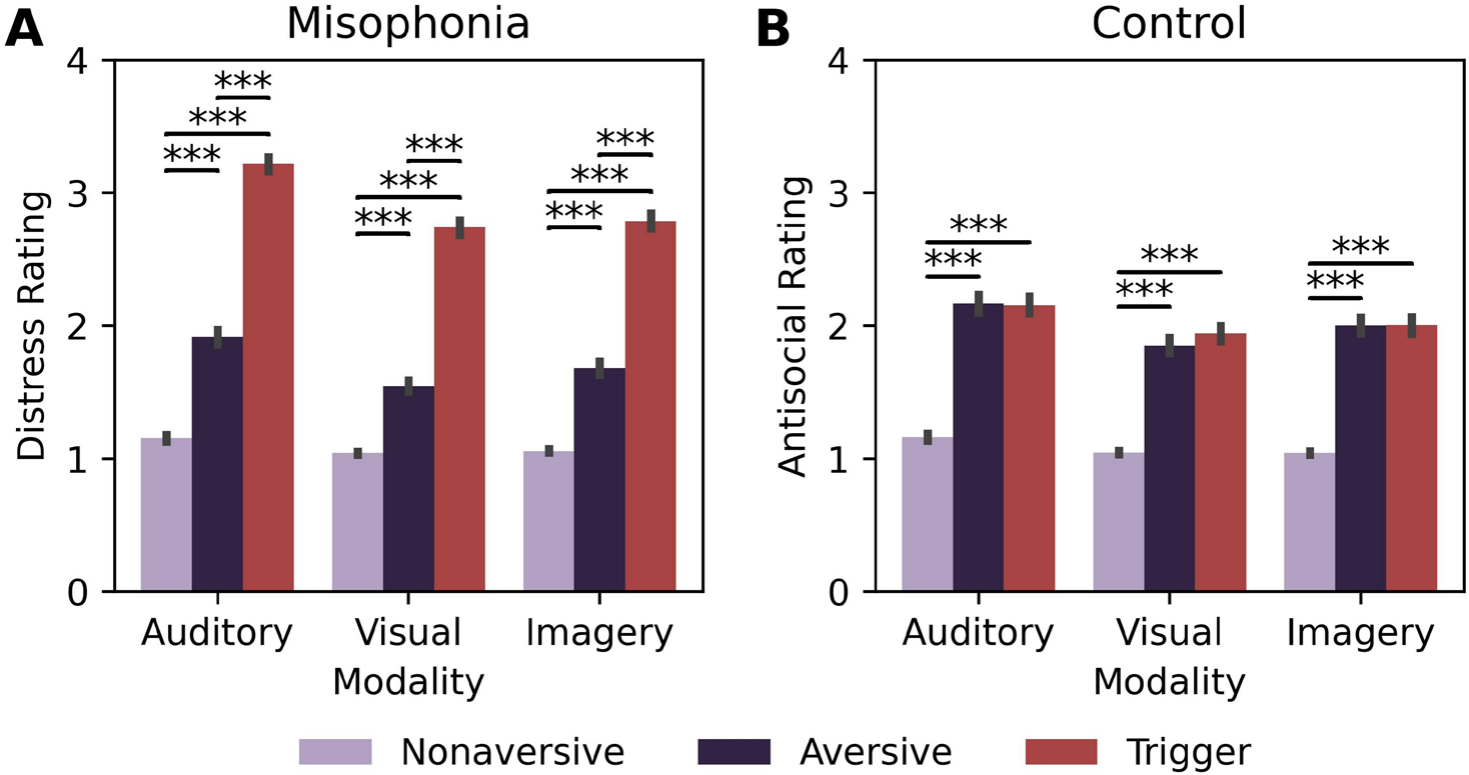
Bar graphs of self‐reported ratings by modality (auditory, visual, imagery) and stimuli condition (non‐aversive, aversive, trigger). (A) Participants with misophonia rated the extent to which the stimuli were distressing. (B) Control participants rated the extent to which the stimuli were antisocial. ***p < 0.001.

For the distress ratings of participants with misophonia, our planned tests showed that trigger stimuli were perceived as significantly more distressing than aversive stimuli for all three modalities, *p*s < 0.001 (Figure 2). Post hoc tests showed that both trigger and aversive stimuli were perceived as significantly more distressing than non‐aversive stimuli for all three modalities, *p*s < 0.001, after applying FDR correction (Figure 2).

For the antisocial ratings of control participants, our planned tests showed no significant differences between trigger and aversive stimuli for all three modalities, *p*s > 0.05 (Figure 2). Post hoc tests showed that, across all three modalities, both trigger and aversive stimuli were perceived as significantly more antisocial than non‐aversive stimuli, all FDR‐corrected *p*s < 0.001 (Figure 2).

### 3.3 Physiological Overactivation to Trigger Relative to Aversive Stimuli in Misophonia Compared With the Control Group (H2)

For the trial‐wise analysis, we examined the simple Group (misophonia vs. control) × Condition (trigger vs. aversive) interaction within each modality. For the auditory modality, participants with misophonia showed significantly greater trigger–aversive differentiation than control participants when listening to trigger sounds characterized by significantly heightened EMGc, EMGz, EDA, and HR responses (Table 2), indicating robust physiological overactivation in misophonia for trigger sounds beyond general aversiveness. No significant difference was found for fingertip SKT.

**TABLE 2:** Planned tests on the simple interaction effects for the trigger‐aversive contrast between misophonia and control groups by modality.

Modality	Channel	Estimate	SE	CI (95%)	*t*	Cohen's *d*	*p*	*p*_FDR_
Auditory	**EMGc**	**0.03**	**0.01**	**[0.02, 0.05]**	**4.02**	**0.27**	**< 0.001**	**< 0.001** ***
	**EMGz**	**0.04**	**0.01**	**[0.02, 0.06]**	**3.87**	**0.27**	**< 0.001**	**< 0.001** ***
	**EDA**	**0.13**	**0.04**	**[0.06, 0.21]**	**3.41**	**0.22**	**< 0.001**	**0.001** **
	**HR**	**1.06**	**0.39**	**[0.31, 1.82]**	**2.75**	**0.18**	**0.003**	**0.009** **
	SKT	−0.01	0.02	[−0.04, 0.02]	0.76	−0.05	0.222	0.278
Visual	**EMGc**	**0.03**	**0.01**	**[0.02, 0.05]**	**3.96**	**0.28**	**< 0.001**	**< 0.001** ***
	EMGz	0.01	0.01	[−0.01, 0.04]	1.28	0.09	0.101	0.138
	EDA	0.07	0.04	[−0.01, 0.15]	1.73	0.12	0.042	0.069
	**HR**	**0.85**	**0.40**	**[0.07, 1.62]**	**2.14**	**0.14**	**0.016**	**0.035** *
	SKT	−0.01	0.02	[−0.04, 0.02]	0.65	−0.04	0.259	0.299
Imagery	EMGc	0.01	0.01	[−0.01, 0.02]	0.58	0.04	0.280	0.300
	EMGz	0.02	0.01	[−0.00, 0.04]	1.57	0.11	0.058	0.088
	**EDA**	**0.09**	**0.04**	**[0.01, 0.17]**	**2.31**	**0.15**	0.**011**	**0.026** *
	HR	0.71	0.40	[−0.06, 1.49]	1.80	0.12	0.036	0.068
	SKT	−0.01	0.02	[−0.04, 0.03]	0.43	−0.03	0.333	0.333

*Note:* Age and sex were controlled for. FDR correction was applied across the 15 planned tests. Rows shown in bold indicate effects that remained statistically significant after FDR correction (*p* < 0.05).

*** *p* < 0.001.

** *p* < 0.01.

* *p* < 0.05, one‐tailed.

For the visual modality, participants with misophonia exhibited significantly greater trigger–aversive differentiation than control participants when watching silent trigger videos, characterized by heightened EMGc and accelerated HR responses (Table 2). EDA responses showed a similar pattern; however, this effect did not survive FDR correction (uncorrected *p* = 0.042; FDR‐corrected *p* = 0.069). No significant effects were observed for EMGz and SKT.

For the auditory mental imagery modality, participants with misophonia exhibited significantly greater trigger–aversive differentiation than control participants when thinking about the trigger sounds, characterized by heightened EDA responses (Table 2). EMGz and HR responses showed similar patterns; however, these effects did not survive FDR correction (uncorrected *p*s < 0.05 and FDR‐ corrected *p*s > 0.05). No significant effects were observed for EMGc and SKT.

**FIGURE 3:**
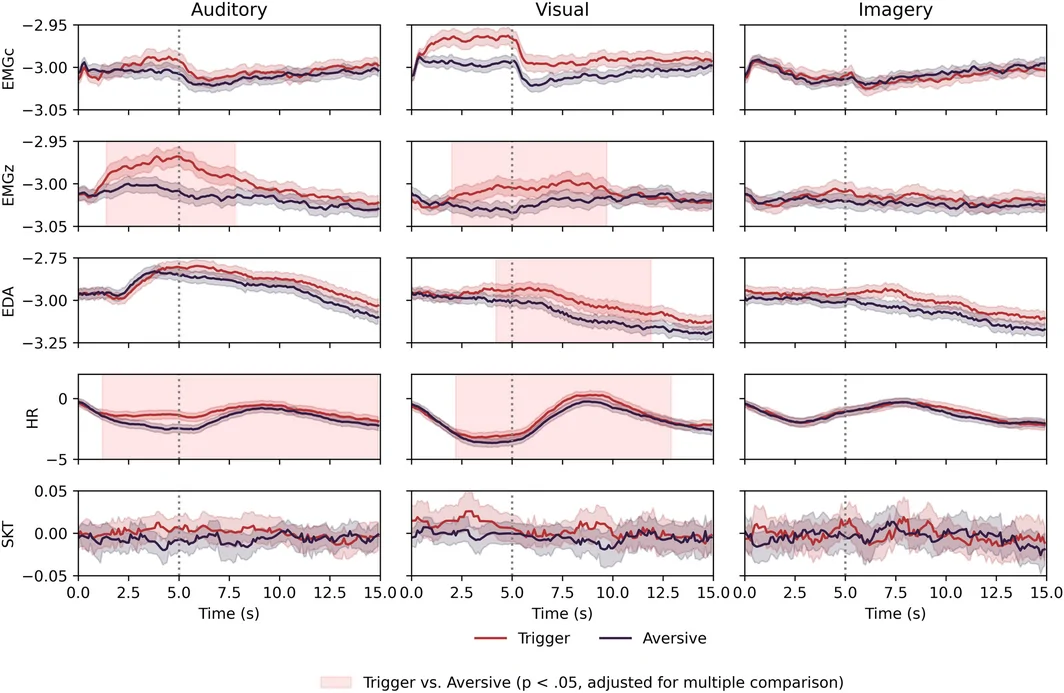
Visualization of signal changes (baseline‐corrected) across time and five physiological channels. Solid lines represent epoch profiles across trials and participants. Bands surrounding the lines indicate the 95% confidence interval. Dotted lines mark the offset time for the 5 s time window of stimulus presentation. Vertical pink shaded regions denote clusters that remained significant after cluster‐based permutation testing (cluster‐wise *p* < 0.05).

To capture fine‐grained differences in the temporal profiles of physiological responses to trigger and aversive stimuli, we conducted epoch‐based analyses in participants with misophonia only (Figure 3). Significant differences between trigger and aversive stimuli were observed at different time windows following stimulus onset for trigger sounds: 1.4–7.8 s for increased EMGz, and 1.2–14.9 s for increased HR (the first column of Figure 3), and for silent trigger videos: 2.0–9.7 s for EMGz, 4.2–11.9 s for increased EDA, and 2.2–12.9 s for increased HR (the second column of Figure 3). Although additional supra‐threshold clusters were observed in other channels and modalities, these clusters did not survive cluster‐based permutation testing.

**FIGURE 4:**
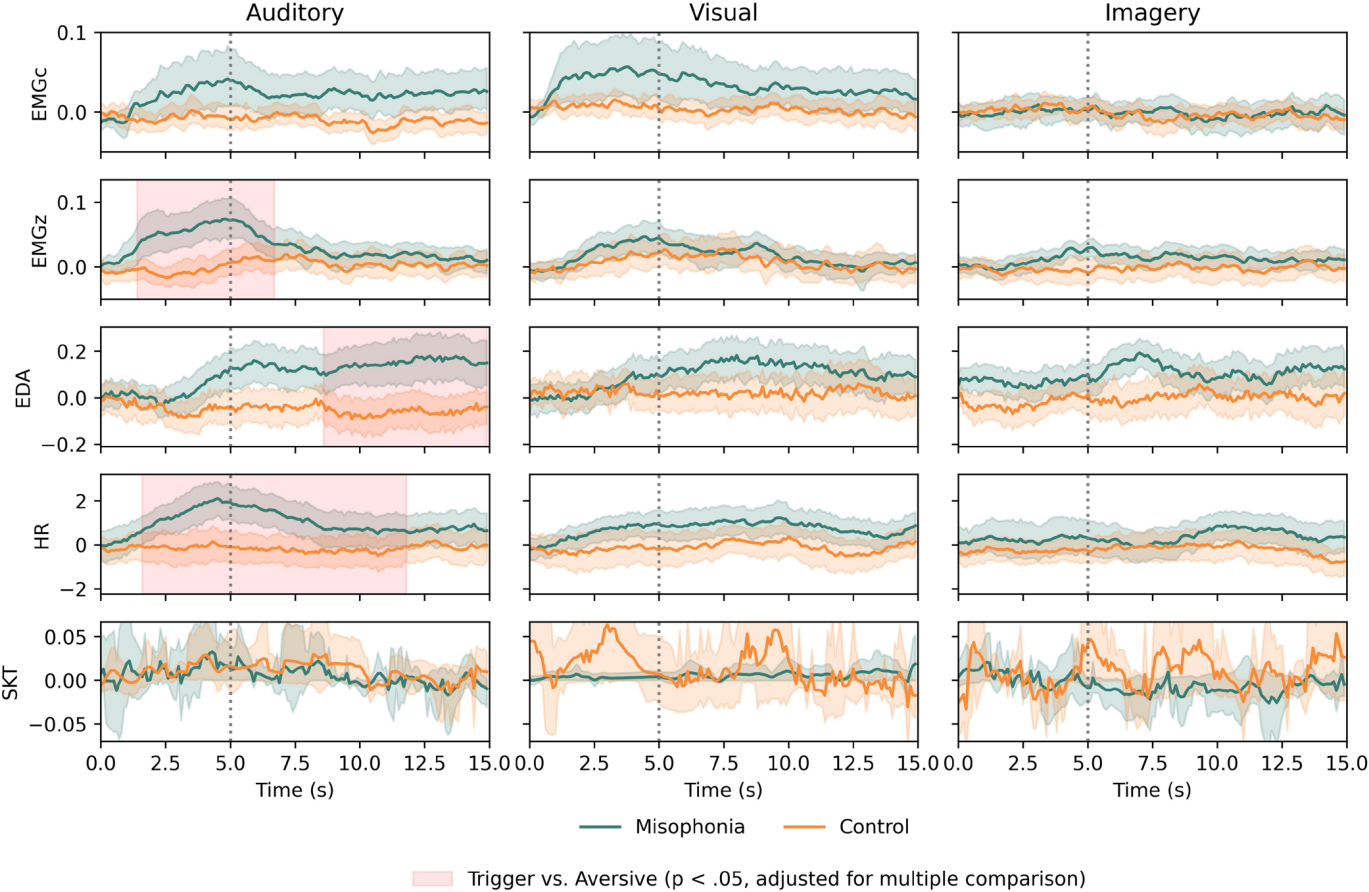
Visualization of signal changes (baseline‐corrected) across time and five physiological channels. Solid lines represent epoch profiles across trials and participants for misophonia (orange) and control (green) participants. Bands surrounding the lines indicate the 95% confidence interval. Dotted lines mark the offset time for the 5 s time window of stimulus presentation. Vertical pink shaded regions denote clusters that remained significant after cluster‐based permutation testing (cluster‐wise *p* < 0.05).

We additionally conducted epoch analyses to examine group differences between participants with misophonia and control participants in physiological overactivation to trigger relative to aversive stimuli (Figure 4). For the auditory modality, significantly greater responses in misophonia relative to control group were observed for EMGz at 1.4–6.7 s, for EDA at 8.6–14.9 s, and for HR at 1.6–11.8 s following stimulus onset (the first column of Figure 4). Although additional supra‐threshold clusters were observed in other channels and modalities, these clusters did not survive cluster‐based permutation testing.

### 3.4 Physiological Activation Modulated by Self‐Reported Distress (H3)

We conducted mixed‐effects GLM analyses to examine whether participants with misophonia exhibit heightened physiological activation to trials that were perceived as more distressing. Supporting our hypotheses, higher distress ratings were positively associated with significantly increased EMGc, EMGz, and EDA for all three modalities (Table 3). Additionally, HR was positively associated with increased distress for auditory and visual modalities only. SKT was not significantly related to distress for any modality.

**TABLE 3:** Planned simple‐slope tests for the effects of self‐reported distress on physiological responses by modality in Misophonia.

Modality	Channel	*β*	SE	CI (95%)	t	Partial *η* ^2^	*p*	*p*_FDR_
Auditory	**EMGc**	**0.020**	**0.002**	**[0.016, 0.024]**	**8.98**	**0.010**	**< 0.001**	**< 0.001** ***
	**EMGz**	**0.019**	**0.003**	**[0.014, 0.024]**	**7.09**	**0.006**	**< 0.001**	**< 0.001** ***
	**EDA**	**0.047**	**0.009**	**[0.029, 0.065]**	**5.09**	**0.003**	**< 0.001**	**< 0.001** ***
	**HR**	**0.281**	**0.096**	**[0.093, 0.468]**	**2.93**	**0.001**	**0.002**	**0.002** **
	SKT	0.000	0.002	[−0.004, 0.004]	−0.05	0.000	0.520	0.520
Visual	**EMGc**	**0.024**	**0.003**	**[0.019, 0.029]**	**9.59**	**0.011**	**< 0.001**	**< 0.001** ***
	**EMGz**	**0.017**	**0.003**	**[0.011, 0.023]**	**5.67**	**0.004**	**< 0.001**	**< 0.001** ***
	**EDA**	**0.040**	**0.01**	**[0.020, 0.060]**	**3.85**	**0.002**	**< 0.001**	**< 0.001** ***
	**HR**	**0.380**	**0.108**	**[0.168, 0.591]**	**3.52**	**0.001**	**< 0.001**	**< 0.001** ***
	SKT	0.000	0.002	[−0.005, 0.004]	0.2	0.000	0.419	0.449
Imagery	**EMGc**	**0.010**	**0.002**	**[0.005, 0.015]**	**4.03**	**0.002**	**< 0.001**	**< 0.001** ***
	**EMGz**	**0.010**	**0.003**	**[0.004, 0.016]**	**3.31**	**0.001**	**< 0.001**	**< 0.001** ***
	**EDA**	**0.037**	**0.010**	**[0.016, 0.057]**	**3.54**	**0.001**	**< 0.001**	**< 0.001** ***
	HR	0.138	0.108	[−0.074, 0.349]	1.28	0.000	0.101	0.126
	SKT	−0.001	0.002	[−0.005, 0.003]	0.44	0.000	0.331	0.382

*Note:* Age and sex were controlled for. Rows shown in bold indicate effects that remained statistically significant after FDR correction (*p* < 0.05).

*** *p* < 0.001.

** *p* < 0.01, one‐tailed.

### 3.5 Physiological Overactivation in Response to Triggers Associated With Misophonia Severity (H4)

We conducted mixed‐effects GLM analyses to examine whether physiological overactivation is more pronounced in individuals who reported greater misophonic severity and functional impairment as measured by S‐Five total score across all participants, separately for the three modalities. Higher S‐Five total scores were associated with greater trigger‐related physiological overactivation for EMGc, EMGz, and HR in the auditory modality and for EMGc in the visual modality (uncorrected *p*s < 0.05); however, no associations survived FDR correction across the 15 planned tests (Table S1).

### 3.6 Physiological Overactivation in Response to Auditory Imagery of Triggers Associated With Auditory Imagery Ability (H5)

To examine if the overactivation in response to trigger relative to aversive stimuli during auditory imagery was associated with the auditory imagery ability, we conducted mixed‐effects models in participants with misophonia, separately for different physiological channels, while controlling for age, sex, and the visual mental imagery ability as measured by VVIQ. Contrary to our hypotheses, no significant results were observed for any physiological channel (Table S2).

## 4 Discussion

The present study provides the most comprehensive examination to date of physiological responses to misophonic trigger stimuli across auditory perception, silent visual cues, and auditory mental imagery using five channels of psychophysiological measures and both trial‐wise and time‐resolved analyses. The clearest evidence emerged for auditory trigger sounds. Supporting our hypothesis, relative to generally aversive sounds, trigger sounds elicited greater physiological overactivation in individuals with misophonia than in control participants across facial EMG, electrodermal activity, and heart rate measures. These findings support the view that misophonic responses to trigger sounds involve heightened affective and autonomic engagement beyond responses to generally aversive stimuli. Evidence for non‐auditory modalities partially supported our hypotheses. Silent visual trigger cues produced significant group differences in some physiological channels, whereas auditory imagery produced weaker and less consistent group‐level effects. Across all three modalities, however, physiological responses in individuals with misophonia were closely related to trial‐by‐trial self‐reported distress. In contrast, the results did not support our hypotheses that this physiological overactivation is modulated by misophonia severity as measured by S‐Five total score or auditory mental imagery ability as measured by BAIS‐V during imagery.

Our strongest findings concern auditory trigger sounds. In the trial‐wise analyses, individuals with misophonia showed significantly greater trigger–aversive differentiation than control participants for corrugator supercilii activity, zygomaticus major activity, electrodermal activity, and heart rate. These effects indicate that auditory triggers evoked heightened facial muscle activity, autonomic arousal, and cardiac activation in individuals with misophonia beyond what was observed for generally aversive sounds. Our examination of the physiological overactivation, that is, the contrast of trigger versus aversive stimuli rather than trigger versus neutral or non‐aversive stimuli, is critical because it indicates that the heightened physiological response to trigger sounds cannot be solely explained by general unpleasantness or aversiveness. Instead, the findings suggest that sounds with misophonic relevance evoke a distinct physiological response profile in individuals with misophonia.

The time‐resolved analyses provide complementary information about how physiological responses unfold over time and how the temporal profiles differ between the misophonia and control groups. Overall, distinct temporal profiles emerged across physiological systems. Facial EMG and heart rate differentiated trigger from aversive stimuli relatively early, with trigger sounds eliciting greater EMG responses and reduced heart rate deceleration beginning approximately 1 s after stimulus onset. In contrast, electrodermal activity exhibited a delayed and sustained differentiation that emerged primarily after stimulus offset, consistent with the slower temporal dynamics of electrodermal responding. Interestingly, although heart rate was consistently higher for trigger than generally aversive sounds in participants with misophonia, this differentiation majorly occurred during the initial cardiac deceleration phase rather than during a subsequent acceleration phase. Specifically, trigger sounds attenuated the magnitude of heart rate deceleration relative to aversive sounds, rather than producing heart rate acceleration above the pre‐stimulus baseline. According to the Defensive Cascade Model, the initial cardiac deceleration reflects orienting and enhanced sensory intake, whereas subsequent heart rate acceleration is thought to reflect defensive action preparation (Bradley, Codispoti, Cuthbert, and Lang 2001; Bradley and Lang 2007). Our findings therefore suggest that misophonic trigger sounds primarily modulate the early orienting stage of motivational processing, leading to altered attentional engagement with trigger stimuli.

Together, these results extend prior findings demonstrating heightened heart rate and electrodermal responses to misophonic triggers (Edelstein et al. 2013; Kumar et al. 2017; Siepsiak et al. 2023). Importantly, beyond these autonomic nervous system responses, our findings show that individuals with misophonia also exhibit concurrent potentiation of facial muscle activity mediated by the facial nerve.^5^ In the general population, this constellation of physiological responses is well documented as a hallmark of processing highly arousing aversive stimuli relative to non‐aversive stimuli (Bradley, Codispoti, Cuthbert, and Lang 2001; Bradley and Lang 2007; Lang et al. 1993; Oh et al. 2025; Yang et al. 2026). The presence of these same responses to misophonic trigger sounds relative to generally aversive stimuli in individuals with misophonia indicates that misophonia is characterized by exaggerated engagement of affective and peripheral physiological systems rather than abnormal sensory processing alone. This interpretation aligns with self‐reported findings showing that individuals with misophonia exhibit markedly reduced tolerance and heightened distress to trigger sounds, consistent with an underlying mechanism of physiological overactivation (Rouw and Erfanian 2018).

The visual modality findings extend the evidence for misophonia by demonstrating that visual cues associated with trigger sounds can also engage misophonic responses, albeit to a lesser extent than auditory triggers. Silent visual trigger cues elicited significantly greater self‐reported distress and trigger–aversive differentiation in individuals with misophonia than in control participants for corrugator supercilii activity and heart rate, while electrodermal activity showed a weaker effect that did not survive FDR correction. These findings suggest that visual representations of trigger‐related events can engage selected components of the misophonic physiological response, particularly facial affective responding and cardiac activation. However, these effects from the trial‐wise analysis in the visual modality were detectable in fewer channels than in the auditory modality, and the time‐resolved group difference analyses did not reveal significant clusters after correction for the visual modality. This pattern of more limited physiological activation is not unexpected. During informal post‐experiment debriefing, 29 participants with misophonia reported that silent trigger videos elicited weaker responses than the corresponding auditory stimuli. Several participants further noted that their reactions depended on specific visual features, such as clearly seeing food being chewed, or suggested that longer video presentations would have been more triggering. These qualitative reports suggest that misophonic responses to visual cues may be more heterogeneous and dependent on the salience and duration of visual cues. Importantly, across both auditory and visual modalities, greater trial‐wise self‐reported distress corresponded to greater corrugator supercilii activity, zygomaticus major activity, electrodermal activity, and heart rate, suggesting that trigger visual cues may engage overactivated physiological responses when they are sufficiently salient to elicit subjective distress. Our results help reconcile the hypothesized misophonic response with the mixed findings in prior literature on visual triggers. The consensus definition of misophonia recognizes that misophonia is characterized by trigger responses not only to sounds themselves but also to other sensory cues associated with those sounds (Swedo et al. 2022). Despite this, few empirical studies have examined misophonic responses to the visual components of auditory triggers. Dozier and Morrison (2017) investigated this question using self‐reported measures and found that visual cues associated with trigger sounds could evoke self‐reported physical reactions similar to those elicited by auditory triggers. In contrast, Edelstein et al. (2013) conducted the only prior psychophysiology study with the visual components of triggers and reported that silent videos of trigger‐related actions did not elicit the same heightened skin conductance responses observed during auditory or audiovisual trigger presentations, leaving the physiological mechanism of visual triggers unresolved. The present results provide evidence that visual cues can evoke measurable physiological differentiation in misophonia.

Our findings regarding the auditory mental imagery were more exploratory, with less consistent group differences across physiological channels and smaller effect sizes. Although auditory imagery of trigger sounds was rated as distressing by individuals with misophonia, group differences in physiological trigger–aversive differentiation were limited. In the trial‐wise analyses, electrodermal activity showed significant group differentiation after correction, whereas effects in other channels were weaker or did not survive FDR correction. The time‐resolved group analyses did not provide robust evidence for imagery‐related group differences. These findings were also not unexpected, given that most participants reported being least triggered by the imagery modality compared with the auditory and visual modalities. Several features of the imagery task may have contributed to the relatively weak group‐level effects. The imagery prompts were brief and relatively simple, and participants were given only five seconds to generate the imagined sound. Affective imagery paradigms often produce stronger physiological responses when scripts are more vivid, personally relevant, response‐focused, and extended in duration (Bradley et al. 2023; Carroll et al. 1982; Lang et al. 1983; Schwartz et al. 1976; Yang et al. 2026). In the present study, participants were presented with brief, generic imagery prompts (e.g., *a woman's high‐pitched scream* or *someone chewing with an open mouth*) for only 5 s. These prompts described only the sound‐producing event and did not provide additional contextual or sensory details to facilitate vivid mental reconstruction. Consequently, participants may not have had sufficient time to generate detailed, personally salient mental imagery capable of eliciting robust physiological overactivation (Yang et al. 2026). In particular, the broad absence of significant fingertip skin temperature decreases may reflect insufficient time for slower peripheral vasoconstrictive responses to emerge. Likewise, the lack of significant associations between physiological overactivation and individual differences in auditory imagery ability may be attributable to the relatively weak imagery manipulation rather than the absence of a relationship between imagery ability and misophonic responding.

Despite these constraints, the present findings remain theoretically and clinically informative. Within the misophonia group, auditory imagery elicited significant self‐reported distress and measurable physiological differentiation between trigger and generally aversive stimuli in selected physiological channels (increased corrugator activity, zygomaticus activity, and electrodermal activity), although these responses were less pronounced than those elicited by direct auditory or visual trigger cues. Relatedly, the absence of reliable heart rate differentiation suggests that the imagery manipulation was sufficient to engage affective and facial motor systems, as reflected in electrodermal and EMG responses, but may not have been sufficiently vivid or immersive to evoke the broader autonomic response observed during direct sensory exposure (e.g., heart rate differentiation). This graded response profile suggests that auditory imagery may provide a useful intermediate level of emotional engagement, activating misophonia‐related affective processes while eliciting weaker physiological responses than direct trigger exposure. Consequently, auditory imagery may represent a promising avenue for interventions involving emotion regulation training or gradual exposure, although future studies using more vivid and contextually rich imagery paradigms are needed to evaluate its therapeutic potential.

Several limitations of the present study should be acknowledged. First, some participants with the most severe misophonia symptoms who could not tolerate the 5‐s stimulus presentation were not recruited. Consequently, the present sample may underrepresent individuals at the extreme end of symptom severity, which may be one reason why we did not observe significant correspondence between physiological overactivation and misophonia severity scores. Second, our self‐selected sample was predominantly female in both groups. Female‐predominant samples have been similarly reported in the self‐selected and treatment‐seeking samples from previous misophonia studies (Ferrer‐Torres and Giménez‐Llort 2021; Guzick et al. 2023; Rouw and Erfanian 2018; Siepsiak et al. 2023; Woolley et al. 2025). Sex‐related differences in psychophysiological responding have also been reported in general samples (Bradley, Codispoti, Sabatinelli, and Lang 2001). In our study, the comparable sex distribution across groups makes it less likely to explain the between‐group effects, but the small number of male participants limits generalizability and the assessment of sex‐specific effects within the misophonia group. Future studies would thus benefit from more sex‐balanced samples. Third, following an established paradigm (Kumar et al. 2017), participants with misophonia rated distress, whereas controls rated perceived antisociality. Although these ratings were chosen to capture behaviorally meaningful responses in each group and were not treated as directly comparable measures, the different rating instructions may have induced different attentional or evaluative states and could therefore have contributed to between‐group differences in task‐evoked responses. Importantly, however, this measurement issue would not necessarily be resolved simply by asking both groups to rate distress using the same conventional Likert scale, because the meaning of a labeled scale and the experiential intensity corresponding to a given label may differ systematically between individuals with and without misophonia. Recent work has highlighted this problem for between‐group comparisons of emotional intensity and proposed psychophysical approaches that anchor judgments to a more general experiential reference, such as the general Labeled Magnitude Scale (Chituc and Scholl 2025). Future studies could therefore use the same rating task across groups while incorporating such psychophysical calibration methods to improve the comparability of subjective reports. Fourth, all analyses were correlational in nature and therefore do not permit causal inferences. Finally, we analyzed each of the five physiological channels independently, whereas physiological responses are inherently multivariate and may be more informative when considered jointly. Consistent with this possibility, prior work has shown that misophonic responses in the visual and auditory imagery modalities can be reliably identified by machine learning models that integrate response patterns across multiple physiological channels (O'Reilly et al. 2025). Future studies may therefore benefit from adopting multivariate analytical approaches to better capture the unique physiological signature of misophonic responses.

In conclusion, the present study provides robust evidence that auditory misophonic trigger sounds elicit heightened physiological responses in individuals with misophonia relative to generally aversive sounds and relative to control participants, supporting the view that misophonia is characterized by heightened affective‐autonomic responding to personally salient trigger events. The findings also suggest that non‐auditory representations of trigger events can engage selected components of this affective‐autonomic response, particularly for silent visual cues and imagery prompts that were experienced as more distressing.

## Author Contributions

**Douglas H. Wedell:** conceptualization, funding acquisition, writing – review and editing. **Xuan Yang:** writing – original draft, investigation, methodology, visualization, writing – review and editing, formal analysis, data curation. **Sewon Oh:** investigation, writing – review and editing, formal analysis, data curation, project administration. **Svetlana V. Shinkareva:** conceptualization, funding acquisition, writing – review and editing, project administration, supervision.

## Funding

This work was supported by a grant from the Misophonia Research Fund.

## Ethics Statement

This study was approved by the Institutional Review Board of the University of South Carolina. All participants provided informed consent in accordance with institutional guidelines.

## Conflicts of Interest

The authors declare the following financial interests which may be considered as potential competing interests: Svetlana Shinkareva and Douglas Wedell report financial support for this research provided by the Misophonia Research Fund. Other authors declare that they have no known competing financial interests or personal relationships that could have appeared to influence the work reported in this paper.

## Supporting information


**Table S1:** Planned tests of the interaction between S‐Five total score and the trigger–aversive contrast by modality for all participants.
**Table S2:** Planned tests of the interaction between BAIS‐V score and the trigger–aversive contrast for the mental imagery modality.

## Data Availability

All auditory and visual stimuli can be found at Misophonia Audiovisual Trigger Archive (MATA) https://osf.io/e7qbm/. All scripts and mental imagery prompts can be found at https://github.com/rayxuanyang/REAM_Psychophysiology. Physiological data will be made available upon request.

## Endnotes

^1^
A sampling rate of 200 Hz yields a Nyquist frequency of 100 Hz, which is insufficient to capture the EMG frequency spectrum, as substantial EMG signal power lies above 100 Hz.

^2^
We have run additional sensitivity analyses while retaining all trigger trials, including those trials that failed to trigger misophonic response and were rated as “not distressing at all,” using the same one‐tailed tests and FDR correction. The overall pattern of findings was highly similar with and without the trial‐exclusion procedure. The great majority of effects that survived FDR correction remained significant in the analyses without the trial‐exclusion procedure. The exceptions were the group‐difference effects for HR in the visual modality and EDA in the imagery modality, as well as the group‐difference effect for EMGz during 1.4–6.7 s for the auditory modality in the epoch‐based analysis.

^3^
Considering that participants with misophonia and control participants did not differ on AIM and ERQ, we did not include these measures in the model.

^4^
We initially used a maximal subject‐level random‐effects structure including random slopes for the within‐subject factors condition and modality and their interaction. Because this structure resulted in singular fits for all physiological measures, we sequentially simplified the random‐effects structure, by including a random slope for condition, which still returned singular fits for HR and SKT. Therefore, for consistency across physiological channels, we only included a random intercept in the final model.

^5^
To explore whether misophonic responses are detectable in facial expressions, we used OpenFace to automatically extract facial action unit values and trained a support vector machine classifier to distinguish trigger from aversive conditions in participants with misophonia with available video coding data (*N* = 38) using leave‐one‐participant‐out cross‐validation. The classifier achieved significantly above‐chance accuracy for the auditory modality (accuracy = 0.524, *p* = 0.031), but not for the visual modality (accuracy = 0.488, *p* = 0.801) or the auditory imagery modality (accuracy = 0.510, *p* = 0.239). Our results suggest that electromyography is sensitive to subtle facial muscle potentiation that may not be readily detectable from facial expression alone.
