# Supplementary material for: Physiological Responses to Auditory, Visual, and Auditory Imagery Triggers in Misophonia
Source: Psychophysiology. 2026 Sep 2;63(9):e70390. doi: 10.1111/psyp.70390 (PMC13537026; doi:10.1111/psyp.70390)
Supplement: Supplementary file 1 [file PSYP-63-e70390-s001.docx]

**Table S1. Planned tests of the interaction between S-Five total score and the trigger–aversive contrast by modality for all participants**

| **Modality** | **Channel** | ***Estimate*** | ***SE*** | ***t*** | ***p*** | ***p_FDR_*** |
| --- | --- | --- | --- | --- | --- | --- |
| Auditory | EMGc | 0.000 | 0.000 | 2.7 | .004 | .053 |
|  | EMGz | 0.000 | 0.000 | 1.74 | .041 | .150 |
|  | EDA | 0.001 | 0.000 | 1.44 | .074 | .160 |
|  | HR | 0.008 | 0.004 | 1.98 | .024 | .120 |
|  | SKT | 0.000 | 0.000 | 0.95 | .172 | .258 |
| Visual | EMGc | 0.000 | 0.000 | 2.41 | .008 | .060 |
|  | EMGz | 0.000 | 0.000 | 0.06 | .476 | .511 |
|  | EDA | 0.001 | 0.000 | 1.26 | .103 | .193 |
|  | HR | 0.007 | 0.004 | 1.65 | .050 | .150 |
|  | SKT | 0.000 | 0.000 | 0.54 | .294 | .365 |
| Imagery | EMGc | 0.000 | 0.000 | -0.21 | .582 | .582 |
|  | EMGz | 0.000 | 0.000 | 1.10 | .136 | .227 |
|  | EDA | 0.001 | 0.000 | 1.51 | .065 | .160 |
|  | HR | 0.002 | 0.004 | 0.58 | .282 | .365 |
|  | SKT | 0.000 | 0.000 | 0.48 | .316 | .365 |

*Notes*. *p*^***^ < .001, *p*^**^ < .01, *p*^*^ < .05, one-tailed. Age and sex were controlled for. The model for SKT in the imagery modality yielded a singular fit warning, indicating a zero random-effect variance. No singular fit warnings were observed for SKT in the auditory or visual modalities, or for any other physiological measures.

**Table S2. Planned tests of the interaction between BAIS-V score and the trigger–aversive contrast for the mental imagery modality**

| **Channel** | ***Estimate*** | ***SE*** | ***t*** | ***p*** | ***p_FDR_*** |
| --- | --- | --- | --- | --- | --- |
| EMGc | 0.000 | 0.000 | -0.52 | .700 | .846 |
| EMGz | 0.000 | 0.000 | 0.24 | .406 | .846 |
| EDA | 0.000 | 0.002 | 0.10 | .461 | .846 |
| HR | -0.018 | 0.017 | -1.02 | .846 | .846 |
| SKT | 0.000 | 0.000 | -0.99 | .838 | .846 |

*Notes*. *p*^***^ < .001, *p*^**^ < .01, *p*^*^ < .05, one-tailed. Age, sex, and VVIQ were controlled for.
